# Aortic Valve Surgery: Fix the Valve or Use a New One?

**DOI:** 10.3390/jcm11164844

**Published:** 2022-08-18

**Authors:** Ilias P. Doulamis, Athanasios Rempakos, Eric W. Etchill, Alexandros Briasoulis

**Affiliations:** 1 Department of Surgery, Johns Hopkins University School of Medicine, Baltimore, MD 21205, USA; 2 Department of Clinical Therapeutics, Alexandra Hospital, Faculty of Medicine, National and Kapodistrian University of Athens, 11528 Athens, Greece; 3 Division of Cardiovascular Medicine, Section of Heart Failure and Transplantation, University of Iowa Hospitals and Clinics, Iowa City, IA 52242, USA

Surgical replacement of the diseased aortic valve (SAVR) has been implemented for over half a century as the surgery of choice to prolong the lifespan of this population of patients [1]. However, both mechanical and biological implanted valves carry several significant risks. Replacement of the native aortic valve with a mechanical one is associated with the need for lifelong anticoagulation, significantly increasing the patient’s risk of hemorrhage. On the other hand, biological valves are more prone to structural deterioration, requiring reoperation. Patients with prosthetic valves are also at risk of prosthesis-related complications, including endocarditis, thromboembolism, and reoperation [2]. Due to these limitations of the replacement modalities, the focus of the cardiac community is now directed on repair techniques as well.

It has only been a couple of decades since attempts have been made to surgically repair the leaking aortic valve [3]. One of the first important milestones of aortic valve repair (AVr) was the preservation of the normally functioning aortic valve in the context of aortic root pathology [4]. Since then many techniques for AVr have been developed, including central cusp plication, free margin resuspension, and pericardial patch repair. Furthermore, an aortic insufficiency classification system has been introduced, which can be used to assess a patient’s eligibility for AVr [5] AVr is a promising technique, which is not without risks. Those primarily stem from the fact that there is lack of expertise in the surgical technique, which involves various technical and surgical challenges due to the anatomy of the aortic valve [6].

Over the years, a wide range of repair techniques as well as biological and mechanical grafts have been studied. However, most of those cohorts have been single-center, retrospective, and non-randomized. Additionally, past studies have focused solely on SAVR or AVr, without comparing the two in the same cohort. Wong et al. published an interesting meta-analysis in 2019, which directly compared the two techniques using the results of eight studies. The study demonstrated no significant difference between SAVR and AVr in the in-hospital mortality, as well as, the 1-year mortality of patients. The reoperation rate at 1 year was found to be higher in patients undergoing AVr, an observation not seen in previous studies. This result was assumed to occur due to the inclusion of smaller studies from centers without great experience in AVr, potentially signifying the aforementioned risks involved in AVr as a result of suboptimal operation technique [7]. 

Taking into account the favorable outcomes of AVr, demonstrated by a variety of studies, patients and physicians now have a decision to make when it comes to the surgical treatment of aortic insufficiency. The demographic characteristics of the patients, comorbidities, valve disease pathophysiology, surgical anatomy, and risk profile of each surgical technique are taken into consideration for the identification of the optimal approach for each patient. Although long-term survival is the ultimate outcome of interest, valve-related complications and quality of life without functional limitations often dictate the patient’s preference over a specific modality.

Among others, age has been reportedly a determining factor for opting the ideal surgical approach. Although mechanical valves used to be the gold standard for younger patients given their longer durability, AVr is now emerging as a viable alternative that does not come at the cost of anticoagulation, while also having a much lower thromboembolic risk compared with SAVR [8,9]. Additionally, AVr can be utilized in children, who often have a complex aortic valve anatomy, while their heart is also constantly growing, making replacement of the aortic valve very challenging [10].

This perspective in addition to the recent advances in transcatheter approaches for aortic valve disease such as transcatheter aortic valve replacement (TAVR) provide an appealing alternative with a safe profile and potentially better quality of life. TAVR was originally presented as an option for patients who were not eligible for surgery, but has since become an important management tool in patients with severe aortic stenosis and certain indications, after demonstrating favorable mortality compared with SAVR [1]. TAVR has also been used in inoperable patients with aortic regurgitation, with studies indicating that it could be a feasible alternative to SAVR in such patients [11]. 

In conclusion, for more than 50 years, SAVR has been the first choice for patients with aortic valve pathology. However, the landscape is beginning to change with the introduction and subsequent development of AVr techniques. AVr was designed to improve on many problematic aspects of SAVR, including the need for lifetime anti-coagulation in patients with mechanical valves, the increased risk of thromboembolism, and the various prosthesis-related complications. The most important impediment to AVr, which has not allowed it to become as widely used as mitral valve repair, is the challenging nature of the operation that arises from the complex anatomy of the aortic valve. As surgeons begin to gain more experience with AVr, new prospective randomized cohorts that directly compare AVr with SAVR and TAVR are necessary to further evaluate which modality provides the most favorable outcomes and quality of life in the various groups of patients with aortic valve pathology.
